# Stem Cell Bioprocessing and Manufacturing

**DOI:** 10.3390/bioengineering7030084

**Published:** 2020-07-31

**Authors:** Joaquim M.S. Cabral, Cláudia Lobato da Silva, Maria Margarida Diogo

**Affiliations:** Department of Bioengineering and iBB—Institute of Bioengineering and Biosciences, Instituto Superior Técnico, Universidade de Lisboa, Av. Rovisco Pais, 1049-001 Lisboa, Portugal; claudia_lobato@ist.utl.pt (C.L.d.S.); margarida.diogo@ist.utl.pt (M.M.D.)

The next healthcare revolution will apply regenerative medicines using human cells and tissues. Regenerative medicine aims to create biological therapies or in vitro substitutes for the replacement or restoration of tissue function in vivo lost due to failure or disease. However, whilst science has revealed the biomedical potential of this approach, and early products have demonstrated the power of such therapies, there is a need for the development of bioprocess technology for the successful transfer of the laboratory-based practice of stem cell and tissue culture to the clinic as therapeutics through the application of engineering principles and practices. This Special Issue of *Bioengineering* on “Stem Cell Bioprocessing and Manufacturing” addresses the central role in defining the engineering sciences of cell-based therapies by bringing together contributions from worldwide experts on stem cell science and engineering, bioreactor design and bioprocess development, scale-up, and the manufacturing of stem cell-based therapies.

In the last few years, human pluripotent stem cell (hPSC) derivatives have emerged as promising allogeneic cell therapy products, with amazing potential to treat a wide variety of diseases and a vast number of patients globally. Brian Lee and co-authors [1] addressed various challenges related to the manufacturing of PSCs in large quantities for commercialization, which include bioreactor process development—namely, scalable bioreactor technology for the large-scale manufacturing of high-quality therapeutic PSCs derivatives.

Among the most promising hPSC derivatives, hepatic cell lineages represent a potential cell source, holding great potential for biomedical applications, such as in liver cell therapy, disease modelling, and drug discovery. João Cotovio and Tiago Fernandes [2] assessed the production of different hepatic cell lineages from PSCs, including hepatocytes, as well as the emerging strategies to generate hPSC-derived liver organoids, highlighting their current biomedical applications.

Alongside the development of novel bioreactor configurations for cell therapy manufacturing, efforts have also been undertaken to optimize bioreactor operating conditions. In their study focused on the manufacturing of mesenchymal stem/stromal cell (MSC) therapies, Josephine Lembong and colleagues [3] developed a fed-batch, microcarrier-based process in a Vertical-Wheel system, which enhanced media productivity while driving a cost-effective and less labor-intensive cell expansion process. As another strategy to improve the cost-effectiveness of cell manufacturing processes, Kathleen Van Beylen and colleagues [4] developed a lactate-based model predictive control strategy for cell growth monitoring and the control of cell proliferation, by adapting the feeding strategy based on lactate measurements, while envisaging the reduction in unnecessary costs, a particularly relevant issue in large-scale cell manufacturing.

An additional key aspect towards the successful translation of cell therapy products is the need to use animal origin-free products (i.e., xeno(geneic)-free) for the derivation, expansion, and differentiation of stem cells in order to minimize the risks of animal-transmitted diseases and immune reactions to foreign proteins. In this context, Valentin Jossen and colleagues [5] developed a bioprocess approach for the expansion of adipose-derived stem cells (ASC), targeting autologous therapies by employing xeno- and serum-free culture conditions and testing static, planar (2D), and dynamically mixed (3D) cultivation systems. To this end, the authors compared the donor variability in both culture systems and developed a mathematical growth model to describe cell growth, nutrient consumption, and metabolite production. Following this same trend, Sandra M. Jonsdottir-Buch and colleagues [6] described the successful proliferation of mesenchymal progenitors derived from human embryonic stem cells (hES-MP) using a culture medium supplemented with human platelet lysates. These hES-MP cells are proposed as interesting alternatives to adult MSC, and the authors demonstrated that these cells can be grown using platelet lysates, maintaining similar proliferation and differentiation profiles to those expanded in culture medium supplemented with FBS.

In addition to the increasing demand for large-scale cell manufacturing protocols, there is a critical need to establish potency assays for stem cell therapy products and their derivatives. Katharina M. Prautsch and colleagues [7] developed a strategy to improve the potency of ASC for nerve regeneration through ex vivo stimulation of ASC with nerve growth factor (NGF). The authors found that the secretome from NGF-stimulated ASC promoted significant axonal outgrowth in an in vitro setting. Upon in vivo delivery of these stimulated ASC (on fibrin-hydrogel nerve conduits), there was an enhancement of early nerve regeneration in a sciatic nerve gap-injury. For other cell therapy candidates, efforts have continued towards the development of the most appropriate culture conditions to establish regenerative phenotypes. This is the case for olfactory ensheathing cells (OECs), a promising therapy candidate for neuronal tissue repair. Rachael Wood and colleagues [8] showed that neither dual co-culture nor fibroblast-conditioned media support the regenerative human OEC phenotype, which means that the appropriate priming conditions to drive a regenerative phenotype in human OECs are yet to be determined. 

Experimental culture conditions are critical for the ex vivo expansion and differentiation of stem cells. In fact, variables such as culture supplements, the purity of the initial cell population, the initial cell concentration, and the duration of culture affect the outcome of stem cell cultures and, consequently, the regenerative potential of ex vivo cultured stem cell-derived products. Sam L. Francis and co-authors [9] focused on the manufacturing of human ASC for articular cartilage regeneration. The authors revealed that there is a higher amount of fat tissue, stromal vascular fraction cell count, and overall yield associated with open (arthrotomy) compared to arthroscopic IFP harvest and described a novel framework for the culture time needed to scale-up the manufacturing of these cells based on the harvesting method.

Finally, cell-delivery methods are a key part of regenerative medicine. The delivery of stem cells and their derivatives can be performed as scaffold-free products (e.g., single cell suspension) or combined with polymer scaffolds. Traditionally, cells and biological agents are implanted into the matrix of the scaffold following electrospinning. The study performed by Nasim Nosoudi and colleagues [10] focused on the development of a novel design that simultaneously introduces cells into the scaffold during the electrospinning process. By demonstrating that human ASC can be directly incorporated into the electrospinning process, maintaining a high viability, the authors suggest the potential benefits of this strategy within the tissue engineering field.
